# Socioeconomic factors associated with suicidal behaviors in South Korea: systematic review on the current state of evidence

**DOI:** 10.1186/s12889-022-12498-1

**Published:** 2022-01-18

**Authors:** Nicolas Raschke, Amir Mohsenpour, Leona Aschentrup, Florian Fischer, Kamil J. Wrona

**Affiliations:** 1 School of Public Health, Bielefeld University, Universitätsstraße 25, 33615 Bielefeld, Germany; 2 Institute of Public Health, Charité – Universitätsmedizin Berlin, Berlin, Germany; 3 Institute of Gerontological Health Services and Nursing Research, Ravensburg-Weingarten University of Applied Sciences, Weingarten, Germany

**Keywords:** Suicide, Suicidal ideation, Attempted suicide, Completed suicide, SES

## Abstract

**Background:**

The economic and human costs of suicide to individuals, families, communities, and society make suicide a major public health problem around the world. Suicide rates in South Korea are among the highest in the world. This paper is the first systematic review investigating socioeconomic risk factors for suicidal behaviors (suicidal ideation, attempted suicides, and completed suicides) in South Korea.

**Methods:**

We performed a systematic review in Medline and Web of Science. Empirical studies and peer-reviewed articles on the association between individual socioeconomic factors and suicidal behaviors have been included. A total of 53 studies were included in a descriptive synthesis.

**Results:**

Overall, 35 studies focused on the association between individual socioeconomic factors and suicidal ideation, 16 were related to suicide attempts, while 10 addressed completed suicides. Low income, unemployment, and financial difficulties were identified as risk factors for all suicidal behaviors. Working in precarious conditions, long working hours, self-employment, changes in employment status, shift work/night-time work, and occupational stress were associated with an increased risk for suicidal ideation. Low educational attainment appears to increase the risk for suicide attempts and completed suicide, but the significance of educational attainment on the reporting of suicidal ideation could not be verified. The primary studies were unable to ascertain whether the place of residence impacts on suicidal behaviors.

**Conclusions:**

The results highlight the relevance of socioeconomic factors for suicidal behaviors in South Korea. Governmental social spending must be increased and redirected more efficiently so that the economically most vulnerable groups are financially protected and income inequality does not widen. Furthermore, comprehensive prevention strategies at the community level are needed. Future research needs to focus on identifying vulnerable groups for whom the effects of low socioeconomic status may have particularly serious consequences with regard to suicidal behaviors.

**Supplementary Information:**

The online version contains supplementary material available at 10.1186/s12889-022-12498-1.

## Introduction

Suicidal behavior occurs throughout the lifespan and can affect people of all social classes. While the mortality rates of completed suicides – which is the best documented suicidal behavior – have been decreasing in most developed countries, South Korea has been ranked top among the OECD countries in terms of suicide rates for over a decade [1, 2]. Since the financial crises of the 1990 s, the age-standardized death rate more than doubled due to intended suicides from 1993 to 2016 and overall continued to increase despite some slight declining trends in some years [3, 4]. Lifetime prevalence of suicidal ideation in South Korea was 24.8% [5]. Howeer, the risk of suicidal behaviors differs according to sociodemographic and socioeconomic factors. For example, public health challenges related to suicide have become even more pronounced among young women in the wake of the COVID-19 pandemic in 2020 [6, 7]. With almost one death every 40 min in South Korea, the current suicide statistics represents an “epidemic”. Across all ages, suicide is the fifth leading cause of death in South Korea; and it is the primary cause of death for adolescents. However, also the elderly are still very prone to suicide [8].

Suicidal behavior is a complex multicausal phenomena, determined by the interaction of individual, relationship, community and societal factors [9]. Completed and attempted suicides are often, but not always preceded by suicidal ideation, which can be seen as an important indicator, even though suicidal ideation by itself is not a sufficient predictor for these actions [10, 11]. Suicidal ideation refers to the thoughts about the desire, intent, and method for committing suicide and may be of varying intensity, ranging from occasional fleeting thoughts to rumination about one’s own death and a current plan to committing suicide [12]. The association between suicidality and socioeconomic factors has been an essential part of suicide research for many years [13, 14]. However, The patterns and magnitude of these factors vary between countries [15], with sometimes even heterogeneous results as observed for suicidal behaviors during the financial crisis in Greece [16].

Despite the major impact that suicidal behavior has on families, society and the economy, the treatment rate for mental illness is extremely low in South Korea, indicating that negative perceptions of the society may lead to underreporting [17, 18]. The underlying reasons behind the stigmatization have not yet been sufficiently investigated. However, the values of collectivism derived from Confucianism, in which family relations have great value, are considered to be one of the main reasons [19]. The honor of the family takes precedence over the individual, resulting in South Koreans rarely talking about suicidal behavior in order to save face with their family. Confucian culture also emphasizes individual volition and self-discipline. In South Korea’s high-performance society, it may lead to the false belief that suicidal ideation and mental illnesses can be overcome purely through willpower, and their occurrence is understood as a sign of weakness and personal failure [20].

Socioeconomic factors are one determinant of suicidal behaviors in a range of multiple and complex causes. Until now, evidence which investigates the association between socioeconomic status (SES) on suicidal behaviors in South Korea is available. However, to our knowledge, this is the first systematic review which summarizes the impact of individual socioeconomic factors (occupation, income/finances, education, place of residence, and overall SES) with different forms of suicidal behaviors (suicidal ideation, suicide attempts, and completed suicides) in South Korea.

## Methods

A systematic literature review was performed in the databases Medline [via PubMed] and the Web of Science Core Collection to identify and examine individual socioeconomic risk factors of suicidal behavior in South Korea. We initially planned to also investigate the influence of external sociocultural factors on suicidal behavior. Hence, our search strategy also looks for external sociocultural factors. However, an inclusion of these factors would go beyond the scope of this paper. Therefore, the results in this regard are not part of this paper.

### Study selection

We used the following search algorithm in PubMed: (“Republic of Korea“[Mesh] OR south korea* OR korea* NOT north korea*) AND (suicide ideat* OR suicide think* OR suicidal behav* OR suicide attempt* OR attempted suicide* OR completed suicide*) AND (“Socioeconomic Factors“[Mesh] OR “Social Class“[Mesh] OR “Culture“[Mesh] OR “Ethnology“[Mesh] OR “Social Perception“[Mesh] OR socio* OR cultur* OR inequal* OR geograph* OR unemploy* OR econom*).

In the Web of Science Core Collection we used the following search algorithm: ts= (south korea* OR republic of korea OR korea* NOT north korea*) AND ts= (suicide ideat* OR suicide think* OR suicidal behav* OR suicide attempt* OR attempted suicide* OR completed suicide* OR suicide thought* OR suicide risk* OR suicide rate*) AND ts= (socio* OR cultur* OR inequal* OR geograph* OR unemploy* OR econom* OR social capital OR social stress OR social support OR social class OR income).

To identify appropriate studies, titles and abstracts were screened by at least two reviewers (NR, AM and KW). They appraised the full-texts if inclusion criteria were fulfilled (Table 1). Discrepancies (34 among 203 full-texts) were resolved by discussion between the reviewers. We included all studies that have been published until 01.05.2020. No further studies were identified when screening the reference lists of the studies included. This indicates that the search algorithm used in PubMed and Web of Science proved to be reliable.

**Table 1:** Inclusion and exclusion criteria

Criteria	Inclusion	Exclusion
Population	All current residents of South Korea	Studies that have no connection to current residents of South Korea or where South Korean residents are not separately reported for
Type of studies	Empirical studies, including qualitative, quantitative studies and mixed-method studies, as well as systematic and narrative reviews of qualitative and/or quantitative and/or mixed-methods research	Studies of theoretical nature with no empirical data
Type of articles	All published peer-reviewed articles	Books; all forms of grey literature including conference abstracts, commentaries, presentations, proceedings, regulatory data, unpublished trial data, government publications, dissertations/theses, journalistic interviews, policy reports as well as any other non-scientific or non-published material
Focus of study	Studies focusing on the association between sociocultural and -economic factors and suicidal behavior	All studies focusing on suicidal behavior with no link to sociocultural and -economic factors in South Korea
Outcome measure	Suicidal behavior, including suicidal ideations, attempted and completed suicides according to definition of other scientific literature	Studies with no link to suicidal behavior
Geographical area	Studies focusing on South Korea	Studies focusing on other countries or where data on South Korea are not separately reported for
Publication language	English	All studies published in any other language than English
Date of publication	No limitations	No limitations

### Synthesis of results

To ensure a systematic management of the information, references located through the search were downloaded to the bibliographical software package Citavi 6, which automatically identifies and removes duplicates.

**Fig. 1:**
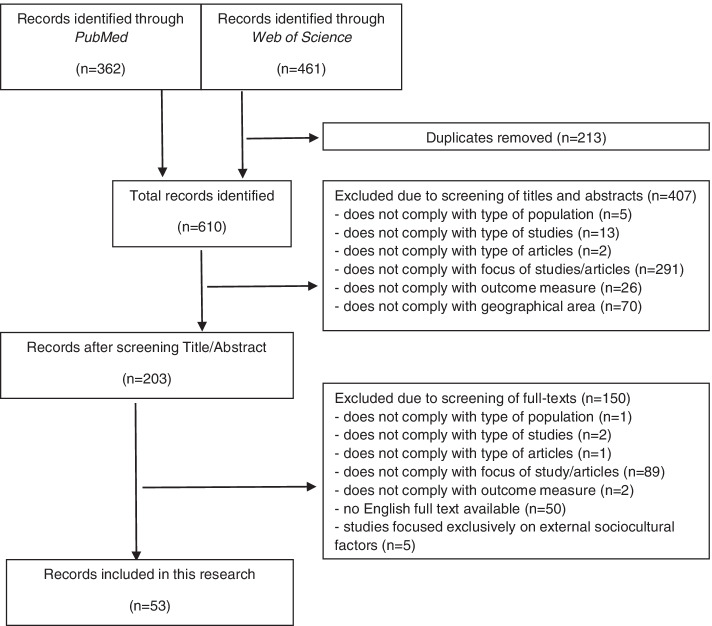
Flowchart of study selection procedure

From a total of 823 hits in two databases, 213 duplicates had to be removed. Overall, 610 titles and abstracts have been screened, leading to 203 articles for full-text review. Among them, 53 studies [5, 21–72] were identified for inclusion in this systematic review (Fig. 1). We synthesized studies according to (1) type of suicidal behavior (suicidal ideation, attempted suicide, and completed suicide) and (2) socioeconomic risk factor (education, occupation, income/finances, place of residence, and overall SES/socioeconomic position).

A descriptive synthesis of the primary studies was performed. Table 2 provides an overview of characteristics and topics covered within the studies.

**Table 2:** Overview of characteristics and topics covered in the included studies (n=53)

	Reference	Type of study	Sample size	Suicidal behavior				Socioeconomic factor				
				**Suicidal ideation**	**Attempted suicide**	**Completed suicide**	**Occupation**		**Income**	**Education**	**Residence**	**SES**
[21]	Ahn et al. 2019	Cross-sectional	22,788	x			x					
[5]	Bagalkot et al. 2014	Cross-sectional	2,964	x	x					x		x
[22]	Chan et al. 2015	Ecological study	197,177^a^			x					x	
[23]	Chin et al. 2011	Cross-sectional	6,969	x			x			x		
[24]	Cho et al. 2007	Prospective cohort study	642,151			x			x			
[25]	Choi et al. 2019	Retrospective cohort study	558,147			x			x			
[26]	Fukai et al. 2020	Cross-sectional	1,200	x					x			
[27]	Han and Lee 2013	Ecological study	5,222	x			x		x	x		
[28]	Hong et al. 2011	Ecological study	83,980	x	x				x			
[29]	Jeong and Chun 2019	Cross-sectional	77,407	x					x	x	x	
[30]	Jo et al. 2015	Cross-sectional	75,238		x		x					
[31]	Joo and Roh 2016	Cross-sectional	543	x			x		x	x		
[32]	Ju et al. 2016	Cross-sectional	58,590	x					x			
[33]	Kang et al. 2015	Cross-sectional	72,623	x	x							x
[34]	Kang et al. 2014	Cohort study	1,204	x			x		x		x	
[35]	Kang et al. 2017	Cross-sectional	14,114	x			x					
[36]	Ki et al. 2017	Pathway analysis, used cross-sectional data	34,565		x		x		x	x		
[37]	Kim et al. 2020	Retrospective cohort study	34,535	x			x					
[38]	Kim et al. 2016a	Cross-sectional	220,245		x		x		x	x		
[39]	Kim and Yoon 2018	Cross-sectional	4,969	x			x		x	x		
[40]	Kim et al. 2006	Case-control study	8,949^a^			x	x		x	x	x	x
[41]	Kim et al. 2016b	Cross-sectional	19,261		x				x	x	x	
[42]	Kim et al. 2017	Retrospective cohort study	2,912	x			x					
[43]	Kim et al. 2010	Ecological study	30,666	x		x				x	x	
[44]	Kim et al. 2014	Path analysis, used cross-sectional data	684	x								x
[45]	Kim and You 2019	Secondary analysis of retrospective panel data	10,988	x	x				x			
[46]	Kim et al. 2018	Cross-sectional	53,969	x			x					
[47]	Kim et al. 2019b	Longitudinal case study	25,862	x			x					
[48]	Kim et al. 2019c	Ecological study	56,151^a^			x	x					
[49]	Ko et al. 2014	Cross-sectional	74,186		x							x
[50]	Kwak and Kim 2017	Cross-sectional	1,431	x			x					
[51]	Lee and Hong 2017	Retrospective study	4,944,632			x			x			
[52]	Lee et al. 2008	Cross-sectional	368	x	x							x
[53]	Lee et al. 2019a	Cross-sectional	247,222		x				x			
[54]	Lee et al. 2017	Retrospective cohort study	1,017,468			x			x			
[55]	Lim et al. 2015	Ecological study	81,354,834			x				x		
[56]	Min et al. 2019	Cross-sectional	64,802	x	x		x					
[57]	Min et al. 2015	Cross-sectional	52,161	x	x		x					
[58]	Moon and Park 2012	Cross-sectional	7,301	x			x		x			
[59]	Park and Lee 2015	Cross-sectional	12,148	x					x	x		
[60]	Park et al. 2016a	Cross-sectional	10,674	x			x		x	x		
[61]	Park and Lee 2016	Cross-sectional	727		x						x	x
[62]	Rim et al. 2020	Cross-sectional	1,212		x					x	x	x
[62]	Ro et al. 2015	Cross-sectional	49,357		x		x		x			
[64]	Shin et al. 2015	Cross-sectional	4,247	x					x			
[65]	Shin et al. 2009	Cohort study	1,857	x					x	x		
[66]	Sohn et al. 2014	Case-control	45,150			x			x			
[67]	Yi and Hong 2020	Cross-sectional	7,257	x			x		x	x	x	x
[68]	Yoon et al. 2015a	Cross-sectional	67,471	x			x					
[69]	Yoon et al. 2015b	Cross-sectional	306	x			x					
[70]	Yoon et al. 2016	Cross-sectional	1,995	x			x					
[71]	Yoon et al. 2015c	Cross-sectional	12,076	x			x					
[72]	Yoon et al. 2017	Cohort study	3,793	x			x					

^a^suicide cases

## Results

Of the 53 studies included in the systematic review, a total of 35 studies dealt with suicidal ideation, 16 with attempted, and 10 with completed suicides. As some studies included multiple definitions of suicidal behavior and socioeconomic factors simultaneously, the sum here is more than 53. Studies were published between 2009 and 2020. All ages and genders were included; the sample size ranged from 168 to 1,025,340 people. A cross-sectional study design was used most frequently, but case-control and longitudinal study designs were also identified. Ecological studies focusing on a population or group level, mostly in the form of time-series-analyses, were included as well. A more detailed overview of the individual articles can be found in Appendices 1 to 4.

### Suicidal ideation

**Fig. 2:**
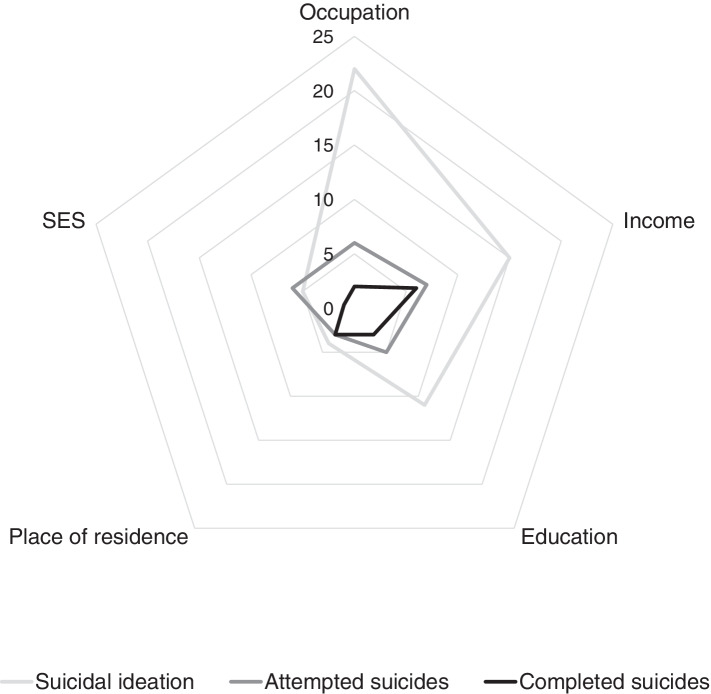
Synthesis of studies related to suicidal behaviors and socioeconomic factors

The most frequently examined socioeconomic factor associated with suicidal ideation (*n*=35) was occupation (*n*=22), followed by income/finances (*n*=15). Further studies investigated education (*n*=11), overall SES/socioeconomic position (*n*=5) and place of residence (*n*=4) (Fig. 2). SI was mainly measured by a dichotomous question, which referred to the past twelve months.

#### Occupation

Twenty-two of the 35 studies examined the relationship between occupation/employment and suicidal ideation. Among these, a total of nine studies investigated the association between unemployment and suicidal ideation. For six of these nine studies, employment status was differentiated into employed or non-employed only. Unemployment was significantly associated (at least in univariate analyses) with suicidal ideation [23, 27, 34, 50, 60, 69]. Three out of these six studies included subjects with at least 65 years of age only, the other three studies included a variation of ages, but focused mainly on the working age population (18–64 years).

Among the three other studies, unemployment was significantly associated with suicidal ideation in the study by Yi and Hong [67], but not in the study by Kim and Yoon [39], whereas Moon and Park [58] observed a higher likelihood for suicidal ideation in relation to unemployment for women only.

Two studies compared the impact between manual and non-manual work on suicidal ideation, one focusing on Korean middle-aged adults [58] and one including participants aged 65 years and older [50]. In both studies, manual workers experienced more suicidal ideation after adjusting for confounding variables.

With regard to various occupational classes and suicidal ideation, it was found in two studies that relative to managers and professional, blue-collar workers (craft/trades workers, machine operators and assemblers, elementary manual workers) and pink-collar workers (clerks, service, and sales workers) were more likely to report suicidal ideation [21, 71]. However, this held not true for male clerks in the study by Ahn et al. [21], in which their odds for suicidal ideation were lower than they were for managers and professionals. Both studies used multivariate logistic regression models to estimate odds rations and adjusted for confounders such as marital status and household income.

Suicidal ideation among self-employed was investigated in two studies [39, 56]. While Min et al. [56] noted that the rate for suicidal ideation was significantly larger within the self-employed (compared to standardly employed) and middle to large business owner were associated with higher odds of reporting suicidal ideation compared to small business owners, no significant difference in the likelihood of suicidal ideation for self-employment status was found in the study by Kim and Yoon [39].

The relationship between changes in job status and its impact on suicidal ideation was examined in three studies [37, 47, 72]. Particularly a negative change was associated with suicidal ideation. In one study, this only applied to men [37].

Shift work or night-time work were significantly associated with suicidal ideation in three studies [35, 68, 71]. In one of these studies, this association was significant in the self-employed/employer group only [68].

Long working hours are related with increased adjusted odds of suicidal ideation [31, 68, 71]. The mediating role of long working hours on suicidal ideation is intensified in combination with other socioeconomic variables, like low SES [68, 71]. In addition to long working hours, precarious working condition increase the risk for suicidal ideation significantly [47, 57, 72].

Occupational stressors associated with suicidal ideation differed by gender: Low job control for men and interpersonal conflict for women remained significant after adjusting for confounders [42, 46, 70].

#### Income/finances

Income or financial difficulties was investigated in 15 out of 35 studies dealing with suicidal ideation. Studies were published between 2009 and 2020 and had between 1,200 and 77,407 participants. Twelve studies considered income per either year or month, while one study focused on income-related inequalities, one examined the association between health expenditure and suicidal ideation, and one evaluated the effect of delayed payment on suicidal ideation.

The outcomes of studies suggest that income significantly influences suicidal ideation in South Korea, because an association was found in all studies. Especially those in the lowest income-classes have unambiguously greater odds for suicidal ideation compared with individuals in high-income classes [26, 27, 29, 32, 34, 39, 58–60, 67]. An income classified as “middle” was associated with lower odds ratios (OR) for suicidal ideation, but the association was not significant in two studies [58, 67]. While two studies found the effect of income on suicidal ideation to be slightly stronger in men [32, 67] opposite results were found as well [26, 58]. Although studies suggest a particular relevance of income for middle-aged participants [39, 59], an association could also be found in studies that included younger individuals [26, 27] and people aged 65 and older [32, 34, 60]. Recent excessive health expenditure [64] and delayed monthly bill payments [45] were found to have the potential to increase the prevalence of suicidal ideation. Parents’ income is not one of the significant childhood predictors related to suicidal ideation [65].

#### Education

11 studies focused on the association between educational attainment and suicidal ideation. The age-range of the participants varied from children (aged 7 years) to 85 years and more. The numbers of participants also differed significantly from each other, the study with the fewest participants included 543 people, the study with the most participants 77,407. Educational attainment was categorized according to the degree/graduation and the number of educational years. Ten of these studies focused on the educational level of the respondent, whereas one study focused on parents’ educational level. While lower levels of education were associated (at least in a univariate analysis) with a higher risk for suicidal ideation in six studies [23, 27, 29, 31, 43, 59], other studies showed contradicting results [39, 60]. In two studies, educational attainment was not statistically significant [5, 67]. This suggests that the influence of education on suicidal ideation is likely related to other variables, such as age, income, and mental health. Included studies showed that the impact of education on suicidal ideation was dependent on the respective age: Young adults between 18–35 years of age with ”2-year college” (OR=0.16, 95% CI: 0.04–0.66) and “4-year college or higher” education (OR=0.28, 95% CI: 0.09–0.90) had lower risks of suicidal ideations compared to “high-school” education. Furthermore, the age-group 36–55 years reported a higher level of suicidal ideation for respondents with 4-year college education or higher (OR=1.93, 95% CI: 1.09–3.41) compared to those with only high school education [39].

#### Place of residence

The effect of area-level characteristics on suicidal ideation was one of the investigated factors in four studies [29, 34, 43, 67]. All studies measured the influence of the degree of urbanicity on suicidal ideation. However, the results are contradictory. For that reason, no conclusions can be drawn whether the place of residence is associated with suicidal ideation.

#### Socioeconomic status/position

Five studies focused on the relationship between SES and suicidal ideation [5, 33, 44, 52, 67]. Only studies that do not equate socioeconomic status with income were considered. The number of participants varied from 368 to 72,623 individuals; and the ages of 12 years to older than 65 were investigated. All studies used a cross-sectional study design. In four studies, participants completed self-administered questionnaires and had to assess their own socio-economic status and assign them to categories. There is evidence that low SES might play an important role for predicting suicidal ideation across various age groups [5, 33, 44, 67], but the extent of the association (also with regard to age and gender) requires more research defining SES more precisely than in the currently available studies. The significance of SES on depression severity, as shown in a path analysis by Kim et al. [44] suggests that SES, via affecting mental health, indirectly influences suicidal ideation.

### Attempted suicides

A total of 16 studies were identified which addressed the association between socioeconomic factors at the individual level and attempted suicides. The most frequently examined factor was income/finances (n=7), followed by occupation (n=6), and overall SES/socioeconomic position (n=6). Education has been investigated in five studies, place of residence in three (Fig. 2).

#### Occupation

Overall, six studies investigated the relationship between occupation and suicide attempts [30, 36, 38, 56, 57, 63]. Unemployment was significantly associated, directly or indirectly by intensifying anxious or depressed mood, with attempted suicides [36, 38, 63]. The association between employment status and suicide attempts was found to be more salient in men and is dependent on age, with strongest associations among men in the age-group between 19 and 44, and inverted associations among the elderly. Other findings found a significant association of suicide attempts for manual work [36], precarious worker [57], self-employed persons with small businesses [56], and part-time work [30].

#### Income/finances

Six studies examined the influence of income. All of these studies elaborated that a low household income was significantly associated, directly or indirectly, with suicide attempts [28, 36, 38, 41, 53, 63]. These results mostly agree with the findings of suicidal ideation. In one study, low income was a relevant risk factor in men but not in women [41], while in another study, the lowest income level was associated with significantly higher rates in only women between the age of 19 and 44 [38]. Individuals in middle-income classes also mostly faced an increased risk of suicide attempts compared with those in the highest income class, but the association was attenuated compared to the lowest income-group [38, 41]. Through mediating the relationship between depression or other relevant direct risk factors (such as physical illness), income was indirectly linked to suicide attempts in two path analyses [36, 63]. However, the influence of household income on suicide attempts was much less important than other variables, such as perceived academic record or psychological factors.

Delayed monthly bill payment (late payments of utility bills or National Health Insurance) as an early risk factor for suicide attempts was examined in the study by Kim and You [45]. Adults with two or more delayed payment during the last 12 months had 10.99 times increased odds of reporting suicide attempts in comparison to having no late payments. Late payment was even a risk factor when subjects reported one delayed payment only.

Hong et al. [28] not only found that lowest income groups have the highest risk for suicide attempts, they also illustrated an increase in income-related inequalities between 2005 and 2007, which emphasizes the tendency that the magnitude of inequalities regarding income becomes greater over time, potentially leading to more suicide attempts in the future [28].

#### Education

Five studies investigated the influence of educational attainment on suicide attempts; three of them only included adults [36, 38, 41], two covered the age group of adolescents [5, 62]. The level of education was measured by the number of educational years, the educational graduation, or rather the last school the subjects have attended.

While one study could not identify a significant association between education and suicide attempts relating to lifetime suicide attempts [5], no formal- or primary school education, compared to college education, was associated with significantly higher odds for suicide attempts in two studies, after adjusting for covariates [38, 41]. This effect was influenced by gender and age, as younger adults aged 19 to 44 years and women had considerably higher OR’s for suicide attempts within the past 12 months [38, 41]. When comparing college education with middle or high school educational levels, the association was found to be strongly attenuated. Neither mother’s or father’s education was significantly associated with suicide attempts in the study by Rim et al. [62], which compared the risk factors between South- and North-Korean adolescents. Ki et al. [36] observed that educational attainment was found to have the largest indirect effect (through increasing the risk for physical illness and problem drinking) for attempted suicide of all investigated variables such as income or occupation.

#### Place of residence

The effects between place of residence and suicide attempts were investigated in three studies [41, 61, 62]. Adolescents living in a large city were significantly more likely to report suicide attempts compared to those living in small cities or rural areas [61]. On the contrary, another study could not identify an association between place of residence and suicide attempts [62]. Living in rural areas was associated with an increased risk of suicide attempts in both genders (male: OR=1.08; female: OR=1.06) for adults between 19 and 65 years and older [41].

#### Socioeconomic status/position

Six studies considered SES – or socioeconomic position, respectively – as one of the variables under study with regard to suicide attempts [5, 33, 49, 52, 61, 62]. Studies which stated that SES was measured solely based on income are included in the synthesis on income.

In four studies, including adolescents only, a low SES (objective or subjective) was significantly associated with an increased risk of suicide attempts [33, 49, 61, 62]. Subjective perception of the socioeconomic position turned out to be the decisive factor and more important in predicting suicide attempts than objective SES [49]. College students with a low SES were about 69.27 times more in risk of attempting suicide [52]. Contrary to the other studies, SES was not a significant factor in the study by Bagalkot et al. [5]. For adolescents from multicultural families, as well as for adolescents from South Korean families and adolescents from North Korean families, a high SES was significantly associated with higher odds for reporting suicide attempts [33, 61, 62]. Among studies which did not select participants according from origin, a middle level SES was either not significant or decreased the likelihood of suicide attempts.

### Completed suicides

The risk factors for completed suicides were the focus often studies identified within this review. The most frequently investigated socioeconomic factors were income/finances (n=6); education and place of residence were part of three studies each, occupation in two studies and overall SES or socioeconomic position in one study (Fig. 2).

#### Occupation

Two studies examined occupation as a potential risk factor for completed suicides [40, 48]. Both studies included participants at the working age from 15 to 64 years [48] or 20 to 64 years respectively [40].

Overall, the strength of the relationship between a particular occupational group and suicide risk is impacted by age, sex, and time period effects. Working in agriculture, forestry and fishery is associated with the highest risk of suicide among all included occupational groups [48]. Unemployment is linked to a higher likelihood of committing suicide, but it is worth mentioning that other defined occupational groups such as housewives, service workers and workers in stores and/or markets, military service, elementary occupations, agricultural, forestry, and fishing workers, machine operators and assemblers, and craftsmen and related occupations had an even higher risk of suicide death than unemployed individuals [40]. The lowest RR for suicide and suicide by pesticide overall was evident in the group of young men and young women (15–39 years) working as managers and professionals. This significantly differed in the age group from 40 to 64 years [40].

#### Income/finances

Six studies investigated the association between income, respectively finances, and suicide deaths [24, 25, 40, 51, 54, 66]. The results are similar to the findings regarding suicidal ideation. Low income, defined as the average annual insurance premium or classified into income subclasses based on salary, must be considered as an important factor contributing to suicide, as it was found to significantly increase the risk in all studies. The strength of the relationship was shown to be influenced by age and gender. Particularly men and individuals of working age are more vulnerable to the negative effect of low income [25, 54]. High medical care expenditure is a predictor for suicide among adolescents and young adults [66]. In the same income class, wage earners have a lower risk of committing suicide than self-employed [51].

#### Education

Three studies highlighted that individuals with lower levels of educational attainment exhibited higher rates of suicide deaths [40, 43, 55]. College education was linked to the least risk of death by suicide in all considered studies. Differences in suicide mortality between the educational groups grew larger over time and were more salient in men than in women [43, 55].

#### Place of residence

Place of residence and its effect on suicide deaths was examined in three studies [22, 40, 43]. In one study a strong increase in suicides between 1992 and 2016 was found among the elderly living in rural areas. Age- and gender-specific differences can also be determined by the fact that the relationship between rural residence and suicide rates among youth and working-age adults declined over time, more notable among women than men [22]. Metropolitan residence was associated with lower suicide rates and fewer odds for suicide compared to living in rural or urban areas [40, 43]. In contrast, one study observed this for females only, while among men, living in cities showed slightly lower odds for suicide than in metropolitan areas [40]. However, since only three studies examined the association between place of residence and completed suicides – and these studies included place of residence as only one of the investigated socioeconomic variables – these findings are insufficient to draw an unequivocal conclusion.

#### Socioeconomic status/position

Only one study presented that as social class declines, the risk of suicide gradually increases, regardless of gender. Outcomes were controlled for covariates such as marital status, area of residence, and age [40].

## Discussion

The results of the systematic review show that unemployment and low income are major individual risk factors for all suicidal behaviors. Working in precarious conditions (job insecurity, part-time work), long working hours, changes in employment status, shift work and night-time work and occupational stress were associated with an increased risk for suicidal ideation. The included studies provide evidence that the strength of the association between a particular risk factor and suicidal behavior is influenced by age and gender. Thus, the influence of income was shown to be particularly strong for men, and low educational attainment was a risk factor for suicide attempts and completed suicides especially for women and younger adults. Occupational stressors differed between the sexes as well, low job control for men and interpersonal conflict for women were associated with suicidal ideation. Furthermore, low SES appears to increase the risk for suicidal behaviors. Included studies were unable to ascertain whether and to what extent place of residence influenced the three investigated suicidal behaviors. This might be due to the design review, which only considered individual socioeconomic factors, as this allowed only a small number of studies to be eligible. Some studies identified that socioeconomic variables were indirectly associated with suicidal behavior, as they affect the mental and physical health.

The reasons for unemployment and low income contributing to suicidal behaviors are manifold and interdependent. Occupation serves to provide social and financial resources [73]. Unemployed individuals are at risk of facing cessations in social inclusion in everyday life, which consequently can lead to a loss of self-esteem and decreased levels of social standing [74]. Low income reduces access to essential goods, causes chronic stress and unhealthy behaviors closely related to suicide, such as excessive drinking and smoking [28, 75, 76].

In some studies included in this systematic review, income was significantly associated with suicidal behavior in the working-age population only, respectively the influence of income decreased with increasing age [38, 39, 59]. This may be due to the life events and responsibilities associated in the working-age, such as financial support of children and paying parents’ medical bills [77]. However, since most studies consider low income to be a general risk factor, it can be assumed that although age influences the strength of the effect, low income is a weighty predictor of suicidal behavior among all ages. Other studies also concluded that the effects of low income on suicidal behavior are stronger in men, which might be explained by the traditional ideas of Korean society [32, 41, 54, 67].

One reason for the strong association between working in agriculture, forestry and fishery (AFF) or military services and completed suicides might be the convenient access to lethal suicidal methods in this occupational fields (e.g. firearms and pesticides) [48, 78]. Another finding of the analyses between occupational groups and suicidal behavior is that blue collar worker respectively manual workers were more likely to report suicidal ideation compared to white- and pink-collar worker [21, 50, 58]. Of particular note are unskilled manual workers, who represented the largest risk group. This might be due to the more physically demanding work and the precarious working conditions as well as the higher likelihood of part-time work. All of these are factors investigated in this systematic review that were found to significantly increase the risk for suicidal ideation [47, 57, 72].

However, all included studies regarding occupational group and suicidal ideation are cross-sectional. Therefore, causality cannot be established. In addition, the economic crisis in 2008 has led to major changes related to occupation [48, 79]. For that reason, for all studies dealing with employment and occupation, the different data collection periods must be considered. However, self-employment has been observed as a risk factor for suicidal behavior, which may be due to exploitative working circumstances (such as long and irregular working hours), lower health insurance coverage and an exclusion from state welfare programs.

Occupational stressors associated with suicidal intention differed by gender: Low job control and lack of reward for men and interpersonal conflict for women remained significant after adjusting for confounders [46, 70]. These results might be explained by the influence of Confucianism, which shaped the traditional gender roles in South Korea. Accordingly, men serve as the primary wage earners and hold control over their work. Job insecurity could thus negatively affect men’s self-image, and, thereby, suicidal thoughts may arise. Further studies also indicate that Korean men’s job satisfaction is more dependent on (extrinsic) reward [80]. Women tend to be psychologically more susceptible to interpersonal stress, the cause of which they often see in themselves [81]. Job insecurity was found to be particularly a risk factor for head of households, as they are more responsible for supporting their families [42].

Interestingly, compared to attempted suicide and completed suicides, the level of education is hardly decisive for the development of suicidal ideation. One explanation might be that people with higher levels of education have better coping strategies [82]. However, most of the included studies lack an adjustment for determinants that may affect the educational attainment, i.e. support and availability of parents or gender differences in terms of educational opportunities [83, 84].

The studies included in the systematic review were insufficient to conclude whether and to what extent place of residence affects suicidal ideation. On the one hand, this could be due to the relatively low amount of studies and study subjects. On the other hand, studies suggest that not the place or residency, but rather community factors such as availability of social welfare facilities and social cohesion within the neighborhood are more relevant when determining predictors for suicidal ideation [85, 86]. However, regional deprivation levels and specific features of neighborhood environment (air pollution, noise, availability of resources) have not been assessed in the primary studies.

Future research should further focus on contextual factors within high-risk spatial clusters as well as on identifying at-risk groups in order to derive targeted prevention strategies for them, as protection policies for vulnerable populations are much needed to prevent suicidality. Since all socioeconomic variables were studies separately, future studies should examine the interdependence between socioeconomic variables to better assess the effect size of a risk factor. In investigating the relationship between socioeconomic variables and suicidal behavior, research should focus on identifying vulnerable groups in order to derive more concrete recommendations for action by policymakers. Gender- and age-specific differences in the effect of socioeconomic factors on suicidal behavior suggest that further studies are needed that address these differences more specifically, with particular attention to the tension between collectivist Confucianism and the phenomenon of a hitherto unknown burgeoning individualism. Furthermore, suicidal behaviors cannot merely be explained by socioeconomic factors, because suicidality is a complex and multifaceted issue. For that reason, further emphasis needs to be on an extensive range of determinants (e.g. inter- and intra-personal factors; social, political, and natural surroundings), which may also be directly or indirectly impacted by socioeconomic factors, to gain a better understanding of suicidal behaviors.

Overall, the results highlight the need for meaningful suicide prevention in South Korea, which must have two goals: (1) Developing effective strategies to minimize risk factors and support vulnerable groups, and (2) breaking the stigma of suicidal ideation and mental illness as a sign of failure and weakness.

In addition to political and economic policies and changes to access to health resources, communities and municipalities play a critical role in suicide prevention. Effective support within communities can help protect vulnerable individuals from suicide by building and improving social relationships and coping skills. Assistance and counseling centers must be created that reflect and address the specific characteristics of victims of suicidal behavior in a region (e.g. high old-age poverty). Gatekeeper, most of whom are the first point of contact in the health care system, need to be trained to recognize linguistic, behavioral, and situational warning signs and learned to intervene in areas at risk for suicide. With the introduction of the “Standardized Suicide Prevention Program for Gatekeeper Intervention in Korea“, the government has taken an important first step [87]. However, effective community suicide prevention goes beyond the involvement of gatekeepers and includes the collaboration of schools, workplaces, leisure programs and places of worship to meet local needs and resources with regard to prevention strategies. Comprehensive suicide prevention efforts at the community level can help reduce what may be the biggest problem: the stigma surrounding suicidal behavior and mental illness.

### Limitations

Several factors need to be considered when interpreting the result; first of all, related to the primary studies themselves. Foremost, the studies are very heterogeneous. All studies focusing either on suicidal ideation or suicide attempts are based on self-reports. Both selection bias and social desirability may lead to an underestimation. Most studies assessed suicidal ideation only by one dichotomous question (yes/no) rather than by a scale consisting of multiple items, covering severity, duration, and variability of suicidal ideation. Furthermore, the risk factors and suicidality have bidirectional effects: For example, a suicide attempt could also decrease economic productivity, resulting in unemployment and poverty. Therefore, the possibility of reverse causality has to be taken into consideration. Although the adjustment of essential confounders is crucial in this area, many studies were secondary analyses of surveys. For that reason, some important variables for these secondary analyses might be missing. Due to the lack of available data, the majority of studies did not control for mental health variables and the outcomes of studies are difficult to compare with those of studies that did. In addition, most studies only collected information about the participants’ current status of health and did not gather information on participants’ history of mental or physical illness.

In addition, limitations occur due to choices made for the systematic review. The literature search is based on two databases and restricted to articles in English language. Thus, studies published in Korean language and grey literature have not been considered. Since there was no restriction on the year of publication, the studies may come to different conclusions, which should be understood in the light of economic and social transformations over time. In this regard, it is worth mentioning the two economic crises at the end of the 1990 s and in 2008/2009, which may have changed the impact of the risk factors. We did not assess risk of bias of primary studies. The influence of confounders has not been measured uniformly, and it is unsure whether flaws in the design, conduct or analysis of a study may lead to biases.

## Conclusions

For the first time, a systematic review investigated socioeconomic risk factors for suicidal ideations, suicide attempts, and completed suicides in South Korea. The large number of studies allows us to map the current state of evidence in this regard. The primary studies highlighted the public health relevance of suicidal behaviors and its associated risk factors in South Korea. There are already high rates of suicide in South Korea and several studies focusing on its risk factors emphasized that one may assume that the negative effect of e.g. low level of education on suicides will become more pronounced in the future. Therefore, there is a need to counteract stigmas related to suicidal behaviors and mental health issues. Furthermore, to counteract socioeconomic risk factors for suicidal behaviors, such as low income and unemployment, economic security must be improved. For this, financial inequalities must be minimized, as the flexibility of the labor market in the globalization process has created risks that directly affect the socially excluded and economically weaker groups. With current poor social welfare provisions, which are clearly lagging behind other OECD countries, vulnerable groups have a high probability of falling into the trap of poverty and social exclusion.

## Supplementary Information

**Additional file 1.**

## Data Availability

All information is provided in the manuscript and appendices.
