# Supplementary material for: Socioeconomic factors associated with suicidal behaviors in South Korea: systematic review on the current state of evidence
Source: BMC Public Health. 2022 Jan 18;22:129. doi: 10.1186/s12889-022-12498-1 (PMC8765829; doi:10.1186/s12889-022-12498-1)
Supplement: Supplementary file 1 — Additional file 1. [file 12889_2022_12498_MOESM1_ESM.pdf]

Annex 1. Review findings on suicidal ideation summarized by individual socioeconomic factor

Authors	Measure of SI	Findings/Strength of association
**Examined factor: Employment/Occupation**		
(Ahn et al. 2019)	Single question: "Have you ever thought of killing yourself during the past year?"	- "Unskilled manual workers" highest OR for SI in both genders[a]<br>- gender specific differences: higher OR for female "clerks" and "Service and sales workers"[a]
(Chin et al. 2011)	Single question: ''Have you ever seriously thought about committing suicide in the past year?'	- employed vs. unemployed: OR 0.68 (CI= (0.58–0.79)[a2]<br>- gender specific differences: lower OR in men when employed[a]
(Han and Lee 2013)	Single question: "Have you ever seriously thought about committing suicide in the past year?"	- employed vs unemployed: OR 0.62 (CI= 0.46-0.84), [c2]
(Joo and Roh 2016)	Single question whether they have had suicidal thoughts during the past year.	≤5 as reference, 5-8 OR 2.45 (CI=1.15-5.23)[a], >8 OR 2.06 (CI=0.85-4.99)[a]
(Kang et al. 2014)	Questions: "Have you ever felt that you would rather be dead? Have you ever felt you wanted to end it all? Have you ever felt like killing yourself?"	- unemployed vs. employed OR 1.96 (CI=(1.22–3.17) in prevalence analysis [a2]
(Kang et al. 2017)	Single question: "During the past year, have you ever felt like you wanted to die?"	- Men: shift work OR 1.099 (CI=0.824-1.467)[a1]<br>- Women: shift work OR 1.246 (CI=0.821-1.89)[a1]
(Kim et al. 2020)	Single question: "Have you ever thought about wanting to die in the last year?"	- not working vs. working<br>• All OR 1.25 (CI=1.02-1.53)[a1]<br>• Males OR 1.72 (CI=1.12, 2.63)[a1]<br>• Females: NS<br>- changes in job status(all): temporary job OR 1.46 (CI=1.12-1.90)[a3],unemployed OR 1.58 (CI=1.21 - 2.06)[a3], daily job NS, self employed NS<br>- gender specific differences in change in job status: change from full-time job to temporary job, daily job or unemployed only significant in males, for females NS
(Kim and Yoon 2018)	Single question about whether the respondent contemplated suicide in the past 12 months	- Young adults (18 – 35 years): out of labor force vs. waged employment OR 2.99 OR (CI=1.08–8.31), unemployment NS, self-employment NS<br>- Middle-aged adults (36-55 years): NS in all categories
(Kim et al. 2017)	Single question whether they had seriously	- low job security = highest HR for SI

Abbreviations: NS = not significant. a = multivariate/multiple regression (adjusted). b = confirmed in univariate analysis, no multivariate/multiple analyses (unadjusted). c = only confirmed in univariate analysis, NS in multivariate/multiple analysis; 1 = $p < 0.05$, 2 = $p < 0.01$, 3 = $p < 0.001$

Authors	Measure of SI	Findings/Strength of association
	contemplated suicide in the previous year	- intermediate job security NS<br>- gender specific differences:<br>Low job security vs. high job security in males HR 3.25 (CI=1.72 - 6.16)[a1], female HR 1.89 (CI=1.20–2.96)[c1]<br>- risk for SI especially in men who were head of households
(Kim et al. 2018)	Single question: “For the past one year, have you ever thought about killing your self?”	- Males: insufficient job control OR 1.27 (CI=1.03–1.57)[a1], lack of reward OR 1.28 (CI=1.60–1.91)[a3], other NS after adjusting for depression,<br>- Females: interpersonal conflict OR 1.22 (CI=1.05–1.44)[a3], other NS after adjusting for depression
(Kim et al. 2019b)	Single question: “Did you seriously think about committing suicide in the past one year?”	- Employment transition: permanent to permanent as control group, permanent to precarious OR 1.74 (CI=1.29–2.35)[a], permanent to Unemployment OR 1.97 (CI=1.32–2.96)[a], precarious to precarious OR 1.86 (CI=1.21–2.85)[a], precarious to unemployment OR 1.43 (CI=1.05–1.95)[a]<br>- Employment status: precarious vs. permanent OR 1.52 (CI=1.17–1.99)[a], unemployed vs. permanent OR 1.36 (CI=1.05–1.78)[a]
(Kwak and Kim 2017)	Single question: “Did you ever think that you wanted to die during the past year?”	- working status: not working vs. working OR 1.615 (CI=1.179-2.213)[a1]<br>- working type: manual vs. non manual OR 4.269 (CI=0.538–33.892)[a1]
(Min et al. 2019)	Single question: Respondents were asked whether they had contemplated suicide in the 12 months preceding the survey.	-standard employment as reference, small business OR 1.25 (CI=1.15 - 1.35)[a1], middle to large business OR 1.32 (CI=1.09-1.61)[a1]
(Min et al. 2015)	Single question whether the respondent had contemplated dying in the past 12 months	Precarious workers vs. non-precarious workers OR 1.41 (CI=1.28–1.55)[a1]
(Moon and Park 2012)	Single question: “Have you thought seriously about suicide during the past 12 months?”	- Males: NS<br>- Females: non-manual as reference, not employed OR 2.55 (CI=1.13–5.79)[a1], manual OR 2.77 (CI=1.20–6.42)[a1]
(Park et al. 2016a)	Single question: “Have you ever seriously thought about committing suicide after you reached sixty years of age?”	Not working OR 1.72 (CI=1.43-2.07)[c3]
(Yi and Hong 2020)	Single question: “Have you ever seriously thought of suicide in the past 12 months?”	-Male: Regular employment as reference, Irregular and economically inactive NS, unemployed OR 23.83[a3]

Abbreviations: NS = not significant. a = multivariate/multiple regression (adjusted). b = confirmed in univariate analysis, no multivariate/multiple analyses (unadjusted). c = only confirmed in univariate analysis, NS in multivariate/multiple analysis; 1 = $p < 0.05$, 2 = $p < 0.01$, 3 = $p < 0.001$

Authors	Measure of SI	Findings/Strength of association
		-Female: Regular employment as reference, Irregular and unemployed NS, economically inactive OR 4.91[a2]
(Yoon et al. 2015a)	Single Question: “Have you ever been willing to die during the past year?”	- Working conditions: only night/shift work in the self-employed/employer group was significantly associated with suicidal ideation<br>- Long working hours: Proportion of individuals with suicidal ideation increased significantly as working hours increased among both male and female subjects.
(Yoon et al. 2015b)	Korean version of Beck’s 19- item Scale for Suicidal Ideation (SSI)	employed subjects had lower SSI scores t=3.60[3]
(Yoon et al. 2016)	Single question: “Have you felt as though you wanted to die during past year?”	high emotional demand in both gender and low job control in men are related to suicidal ideation in sales and service workers[a1]
(Yoon et al. 2015c)	Single question: “Have you ever been willing to die during the past year?”	- Long working hours, shift work or night work and blue-collar work is related to SI in both gender[a1]
(Yoon et al. 2017)	Single question: “Have you ever seriously thought about dying by suicide in the past year?”	change in employment status from permanent to precarious employment increases the risk of SI[a1]
	**Examined factor: Income**	
(Fukai et al. 2020)	Single Question: “[T]hought that you would be better off dead or of hurting yourself in some way”	- Men: <2 million KRW OR 6.171 (CI=2.296–16.582)[a3]<br>-Women: <2 million KRW OR 6.447 (CI=2.960–14.042)[a3]
(Han and Lee 2013)	Single question: “Have you ever seriously thought about committing suicide in the past year?”	- 2 010 000 KRW or more reduces the risk of SI[significant when considering household-level variables1, NS when considering individual, household and administrative-area levels]
(Hong et al. 2011)	Single question: “Have you ever felt like dying in the past 12 months?”	- persistent pro rich inequalities in suicidal ideation, meaning that the lowest income group had the highest risk for suicidal ideation.<br>- Inequalities slightly increased over time
(Jeong and Chun 2019)	Single question: Presence or absence of SI during the last the past year	- 5 million or more as reference, 2.00 – 2.99 million OR 1.36 (CI=1.21-1.54)[a3], other NS
(Joo and Roh 2016)	Single question whether they have had suicidal thoughts during the past year	<10 as reference, 10-29 OR 0.63 (CI=0.28-1.41)[a], 30-49 OR 0.60 (CI=0.25-1.46)[a], ≥50 OR 0.78 (CI=0.32-1.85)[a]

Abbreviations: NS = not significant. a = multivariate/multiple regression (adjusted). b = confirmed in univariate analysis, no multivariate/multiple analyses (unadjusted). c = only confirmed in univariate analysis, NS in multivariate/multiple analysis; 1 = $p < 0.05$, 2 = $p < 0.01$, 3 = $p < 0.001$

Authors	Measure of SI	Findings/Strength of association
(Ju et al. 2016)	Single question: “Have you thought about killing yourself in the past 12 months?”	- Men: high as reference, middle OR 1.22 (CI=1.03–1.46)[a1], low OR 1.77 (CI=1.44–2.17)[a1]<br>- Women: high as reference, middle OR 1.14 (CI=1.01–1.29)[a1], low OR 1.27 (CI=1.10–1.46)[a1]
(Kang et al. 2014)	Questions: “Have you ever felt that you would rather be dead? Have you ever felt you wanted to end it all? Have you ever felt like killing your-self?”	>200 USD as reference, <200 USD OR 2.39 (CI=1.52-3.75)[a3]
(Kim and Yoon 2018)	Single question about whether the respondent contemplated suicide in the past 12 months.	- Young adults (18 – 35 years): NS<br>- Middle-aged adults (36-55 years): likelihood of suicidal ideation appeared to decrease as income level increased
(Kim and You 2019)	Single Question: “Have you seriously considered suicide (ideation) at any time in the past year?”	- 1 late payment vs. never: OR 1.65 (CI= 1.05–2.55)[a1]<br>- 2 or more vs. never: OR 2.32 (1.39–3.86)[a2]
(Moon and Park 2012)	Single question: “Have you thought seriously about suicide during the past 12 months?”	- Men: NS<br>- Women: high as reference, low OR 1.78 (CI=1.16–2.75)[a1], other NS
(Park and Lee 2015)	Single Question: ‘‘Have you ever seriously considered committing suicide or taking your own life in the past year?’’	- Income level only significant of those 45-64 years, low income OR 1.60 (CI=1.09–2.35)[a1]
(Park et al. 2016a)	Single question: “Have you ever seriously thought about committing suicide after you reached sixty years of age?”	1st quintile as reference, 5th quintile OR 0.59 (CI=0.42–0.83)[a1], other NS
(Shin et al. 2015)	Single question: “Have you seriously considered suicide at any time in the past year?”.	- more recent EHE resulted in more suicidal ideation.
(Shin et al. 2009)	Single question: “I think about killing myself” (last 6 month)	NS
(Yi and Hong 2020)	Single question: ‘Have you ever seriously thought of suicide in the past 12 months?	- Men: low as reference, middle NS, high OR 0.07[a1]<br>- Women: low as reference, middle NS, high OR 0.19[a1]
**Examined factor: Education**		
(Bagalkot et al. 2014)	Scale for Suicide Ideation (SSI by Beck et al., self-rated scale)	NS
(Chin et al. 2011)	Single Question: ‘‘Have you ever seriously thought about committing suicide in the past year?”	- Men: primary or less as reference, high school OR 0.65 (CI=0.42–0.99)[a1], college or more OR 0.56 (CI=0.36–0.88)[a1], other NS<br>- Women: primary or less as reference, high school OR 0.68 (CI=0.51–0.91)[a2], college or more OR 0.46 (0.33–0.64)[a2], other NS

Abbreviations: NS = not significant. a = multivariate/multiple regression (adjusted). b = confirmed in univariate analysis, no multivariate/multiple analyses (unadjusted). c = only confirmed in univariate analysis, NS in multivariate/multiple analysis; 1 = $p < 0.05$, 2 = $p < 0.01$, 3 = $p < 0.001$

Authors	Measure of SI	Findings/Strength of association
(Han and Lee 2013)	Single question: “Have you ever seriously thought about committing suicide in the past year?”	- middle school or below as reference, high school OR 0.62 (CI=0.43-0.90)[c1], college OR 0.50 (CI=0.26-0.94)[c1], university or graduate school OR 0.45 (CI=0.31-0.67)[c3]
(Jeong and Chun 2019)	Single question: Presence or absence of SI during the last the past year	College or more as reference, no formal education OR 1.56 (CI=1.37-1.78)[a3], elementary school OR 1.28 (CI=1.13-1.46)[a3], other NS
(Joo and Roh 2016)	Single question whether they have had suicidal thoughts during the past year	- ≤Elementary school as reference, middle school OR 0.56 (CI=0.25-1.25)[a], high school OR 0.88 (CI=0.38-2.05)[a], ≥college OR 0.57 (CI=0.11, 2.91)[a]
(Kim and Yoon 2018)	Single question about whether the respondent contemplated suicide in the past 12 months.	- Young adults (18-35 years): high school as reference, 2-year college OR 0.16 (CI=0.04–0.66)[1], 4-year college or higher OR 0.28 (CI=0.09–0.90)[1], other NS<br>- Middle-aged adults (36-55 years): 4-year college or higher OR 1.93 (CI=1.09–3.41)[1], other NS
(Kim et al. 2010)	Single Question: ‘‘Have you ever felt like dying in the past year?’’	- Those with lower levels of educational attainment exhibited higher rates of suicide.
(Park and Lee 2015)	Single Question: ‘‘Have you ever seriously considered committing suicide or taking your own life in the past year?’’	- only significant among those younger than 65 years of age.<br>- University graduates as reference, middle school graduates aged 24–44 years OR 3.01 (CI=1.57–5.79)[a1], no education or with an elementary school education aged 45–64 years OR 1.70 (CI=1.16–2.51)[a1], other NS
(Park et al. 2016a)	Single question: “Have you ever seriously thought about committing suicide after you reached sixty years of age?”	- no education as reference, 13 years or more OR 1.87 (CI=1.08–3.23)[a1], other NS
(Shin et al. 2009)	Single question: “I think about killing myself” (last 6 months)	NS
(Yi and Hong 2020)	Single question: ‘Have you ever seriously thought of suicide in the past 12 months?	NS
**Examined factor: Place of residency**		
(Jeong and Chun 2019)	Single question: Presence or absence of SI during the last the past year	- large city as reference, rural OR 1.44 (CI=0.98-2.10, p=0.061), other NS

Abbreviations: NS = not significant. a = multivariate/multiple regression (adjusted). b = confirmed in univariate analysis, no multivariate/multiple analyses (unadjusted). c = only confirmed in univariate analysis, NS in multivariate/multiple analysis; 1 = $p < 0.05$, 2 = $p < 0.01$, 3 = $p < 0.001$

Authors	Measure of SI	Findings/Strength of association
(Kang et al. 2014)	Questions: “Have you ever felt that you would rather be dead? Have you ever felt you wanted to end it all? Have you ever felt like killing your-self?”	- urban OR 2.2 (CI=1.53–3.16)[c3]
(Kim et al. 2010)	Single Question: ‘‘Have you ever felt like dying in the past year?’’	- Consistent patterns of inequalities by degree of urbanicity over time could not be found<br>- age-standardized prevalence of SI by urbanicity varied over the ten-year period
(Yi and Hong 2020)	Single question: ‘Have you ever seriously thought of suicide in the past 12 months?	NS
**Examined factor: Socioeconomic Status/Socioeconomic Position/Social class**		
(Bagalkot et al. 2014)	Scale for Suicide Ideation (SSI by Beck et al., self-rated scale)	High as reference, middle NS, low OR 1.93 (CI=1.12-3.33)[c1]
(Kang et al. 2015)	Single Question: ‘Have you ever seriously thought about committing suicide in the past 12 months?’	- high as reference, middle OR 0.9 (CI= 0.86-0.94)[a3], low OR 1.48 (CI=1.41-1.56)[a3]
(Kim et al. 2014)	Korean version of the Beck Scale for Suicide Ideation (K-BSI)	- SES as significant indirect predictor for SI, mediated by depression severity
(Lee et al. 2008)	Single Question: Any suicidal thoughts within the past two Week?	NS
(Yi and Hong 2020)	Single question: ‘Have you ever seriously thought of suicide in the past 12 months?	- Men: low as reference, middle NS, high OR 0.24[a1]<br>- Women: NS

Abbreviations: NS = not significant. a = multivariate/multiple regression (adjusted). b = confirmed in univariate analysis, no multivariate/multiple analyses (unadjusted). c = only confirmed in univariate analysis, NS in multivariate/multiple analysis; 1 = $p < 0.05$, 2 = $p < 0.01$, 3 = $p < 0.001$

Annex 2. Review findings on attempted suicides summarized by individual socioeconomic factor

Authors	Measure of attempted suicide	Findings/Strength of association
**Examined factor: Employment/Occupation**		
(Jo et al. 2015)	Dichotomous question: "Have you ever attempted suicide during the last 1 year?"	- Middle school students: Part-time work OR 1.59 (CI=1.37-1.83)[a3]<br>- High school students: NS
(Ki et al. 2017)	Dichotomous question: "Have you ever attempted suicide in the last 12 months?"	- Direct effects: manager or office work (ß=0.195, P=.02) and unemployment (b=0.212, P<.01) significantly associated with a higher prevalence of suicide attempts.<br>- unemployment showed largest total effect among all socioeconomic variables (b=0.291, P<.01)
(Kim et al. 2016a)	Dichotomous question whether respondent had attempted suicide in the past 12 months.	- Young adults:<br>• Men: unemployed OR 2.41 OR (CI=1.34-4.34)[a1]<br>• Women: unemployed OR 1.31 (CI=0.91-1.87)[a1]<br>- Middle aged:<br>• Men: unemployed OR 1.41 (CI=0.87-2.28)[a1]<br>• Women: unemployed OR 1.46 (CI=0.91-2.36)[a1]<br>- Elderly:<br>• Men: unemployed OR 0.98 (CI=0.59-1.65)[a1]<br>• Women: unemployed OR 0.78 (CI=0.51-1.17)[a1]
(Min et al. 2019)	Dichotomous question: Respondents were asked whether the respondent had attempted suicide in the 12 months preceding the study.	- standard employment as reference, self-employment small business OR 1.67 OR (CI=1.14-2.45)[a1], middle to large business NS
(Min et al. 2015)	Dichotomous question whether the respondent had attempted suicide in the past 12 months.	Precarious work OR 1.52 (CI=1.02–2.27)[a1]
(Ro et al. 2015)	defined as<br>cases in which the response indicated suicide was actually attempted within the past year.	- no job significantly associated with SI, analyzed via x^2-test<br>- Having no job affects AS through depression and SI.
**Examined factor: Income**		
(Hong et al. 2011)	Dichotomous question whether the respondent had ever attempted suicide in the past 12 months	- pro rich inequalities in suicide attempts (the poorer, the more suicide attempts)<br>- Income-related inequalities increased strongly between 2005 – 2007

Abbreviations: NS = not significant. a = multivariate/multiple regression (adjusted). b = confirmed in univariate analysis, no multivariate/multiple analyses (unadjusted). c = only confirmed in univariate analysis, NS in multivariate/multiple analysis; 1 = $p < 0.05$, 2 = $p < 0.01$, 3 = $p < 0.001$

Authors	Measure of attempted suicide	Findings/Strength of association
(Ki et al. 2017)	Dichotomous question: "Have you ever attempted suicide in the last 12 month?"	- low levels of household income significantly associated with AS; total effect ß=-0.154 (direct ß=-0.106, indirect ß=-0.048)[2]
(Kim et al. 2016a)	Dichotomous question whether respondent had attempted suicide in the past 12 months.	- Young adults:<br>• Men: Q1 OR 1.22 (CI= 0.54-2.78)[a1], Q2 OR 1.81 (CI=0.99-3.29)[a1], Q3 OR 1.8 (CI= 0.99-3.27)[a1], Q4 as reference<br>• Women: Q1 OR 1.96 (CI= 1.08-3.55)[a1], Q2 OR 1.63 (CI= 0.96-2.75)[a1], Q3 OR 1.5 (CI= 0.90-2.50)[a1], Q4 as reference<br>- Middle Aged:<br>• Men: Q1 OR 1.93 (CI= 0.88-4.25)[a1], Q2 OR 1.47 (CI= 0.69-3.10)[a1], Q3 OR 0.89 (CI= 0.40-2.00)[a1], Q4 as reference<br>• Women: Q1 OR 1.53 (CI= 0.79-2.98)[a1], Q2 OR 0.65 (CI= 0.35-1.19)[a1], Q3 OR 1.05 (CI= 0.59-1.87)[a1], Q4 as reference<br>- Elderly:<br>• Men: Q1 OR 1.72 (CI= 0.42-7.08)[a1], Q2 OR 1.31 (CI= 0.29-5.88)[a1], Q3 OR 5.32 (CI= 1.19-23.73)[a1], Q4 as reference<br>• Women: Q1 OR 1.15 (CI= 0.49-2.70)[a1], Q2 OR 0.97 (CI= 0.36-2.59)[a1], Q3 OR 1.53 (CI= 0.50-4.73)[a1], Q4 as reference
(Kim et al. 2016b)	Dichotomous, defined as having experienced SI and actually making a suicide attempt in the past year	- Men: 201-400 million won OR 1.59 (CI=0.97-2.61)[a1], 101-200 million won OR 1.84 (CI=1.12-3.03)[a1], ≤100 million won OR 1.94 (CI=1.14-3.28)<br>- Women: 201-400 million won OR 0.95 (CI=0.69-1.30)[a1], 101-200 million won OR 0.86 (CI=0.62-1.21)[a1], ≤100 million won OR 0.96 (CI=0.66-1.39)[a1]
(Kim and You 2019)	Dichotomous question: "Have you tried to commit suicide at any time in the past year?"	- 1 late payment vs. never OR 5.46 (CI=(1.82–16.39)[a2]<br>- 2 late payments vs. never OR 7.44 (CI=2.89–19.20)[a3]
(Lee et al. 2019a)	Dichotomous question: "In the past year, have you ever attempted suicide?"	- low income OR 1.038 (CI=1.002–1.076)[a1]<br>- low income as one of 14 included for predicting suicide attempts
(Ro et al. 2015)	defined as<br>cases in which the response indicated suicide was actually attempted within the past year.	- Income as indirect factor for AS through mediating the relationship between depression or other relevant direct risk factors such as physical illness and AS
**Examined factor: Education**		
(Bagalkot et al. 2014)	Dichotomous question: "Have you ever attempted suicide?"	NS

Abbreviations: NS = not significant. a = multivariate/multiple regression (adjusted). b = confirmed in univariate analysis, no multivariate/multiple analyses (unadjusted). c = only confirmed in univariate analysis, NS in multivariate/multiple analysis; 1 = $p < 0.05$, 2 = $p < 0.01$, 3 = $p < 0.001$

Authors	Measure of attempted suicide	Findings/Strength of association
(Ki et al. 2017)	Dichotomous question: "Have you ever attempted suicide in the last 12 month?"	- Low levels educational attainment significantly associated with a higher prevalence of AS: total effect ß=-0.282[2], direct effect ß=-0.158[2], indirect effect ß=-0.124[2] (largest indirect effect of all included variables)
(Kim et al. 2016a)	Dichotomous question whether respondent had attempted suicide in the past 12 months.	- Young adults:<br>• Men: primary or less OR 2.41 (CI=1.05-5.56)[a1], middle or high school OR 1.81 (CI=1.17-2.80)[a1]<br>• Women: primary or less OR 5.35 (CI=1.75-16.34)[a1], middle or high school OR 2.2 (CI=1.49-3.26)[a1]<br>- Middle aged:<br>• Men: primary or less OR 1.58 (CI=0.78-3.20)[a1], middle or high school OR 1.32 (CI=0.73-2.39)[a1]<br>• Women: primary or less OR 0.86 (CI=0.48-1.53)[a1], middle or high school OR 0.69 (CI=0.39-1.22)[a1]<br>- Elderly:<br>• Men: primary or less OR 1.39 (CI=0.58-3.36)[a1], middle or high school OR 1.83 (CI=0.77-4.34)[a1]<br>• Women: not covered
**Examined factor: Socioeconomic Place of residence**		
(Kim et al. 2016b)	Dichotomous, defined as having experienced SI and actually making a suicide attempt in the past year	- Men: middle or high school OR 1.22 (CI=0.88-1.71)[a1], primary school OR 1.05 (CI=0.67-1.64)[a1], non-formal education OR 1.14 (CI=0.57-2.28)[a1]<br>- Women: middle or high school OR 1.75 (CI=1.29-2.37)[a1], primary school OR 1.81 (CI=1.19-2.76)[a1], non-formal education OR 2.39 (CI=1.44-3.97)[a1]
(Rim et al. 2020)	Dichotomous question: "Have you attempted suicide during the past 12 months?"	NS
(Kim et al. 2016b)	Dichotomous, defined as having experienced SI and actually making a suicide attempt in the past year	- Men: rural OR 1.08 (CI=0.83-1.42)[a1]<br>- Women: rural OR 1.06 (CI=0.85-1.32)[a1]
(Park and Lee 2016)	Dichotomous question: question: "Have you attempted suicide during the past 12 months?"	- large city OR 9.92 (CI=1.29–76.40)[b1], other NS
(Rim et al. 2020)	Dichotomous question: "Have you attempted suicide during the past 12	NS

Abbreviations: NS = not significant. a = multivariate/multiple regression (adjusted). b = confirmed in univariate analysis, no multivariate/multiple analyses (unadjusted). c = only confirmed in univariate analysis, NS in multivariate/multiple analysis; 1 = $p < 0.05$, 2 = $p < 0.01$, 3 = $p < 0.001$

Authors	Measure of attempted suicide	Findings/Strength of association
	months?"	
**Examined factor: Socioeconomic Status/Socioeconomic Position/Social class**		
(Bagalkot et al. 2014)	Dichotomous question: "Have you ever attempted suicide?"	NS
(Kang et al. 2015)	Dichotomous question: 'Have you ever attempted suicide in the past 12 months?'	- middle OR 0.73 (CI=0.67-0.80)[a3], low OR 1.3 (CI=1.18-1.42)[a3]
(Ko et al. 2014)	Dichotomous question: "During the last 12 months, have you attempted suicide?"	- Adolescents with low levels of both the subjective economic status and objective SEP in the middle school, and with low subjective SEP and high objective SEP in the high school at higher risk of attempting suicide
(Lee et al. 2008)	Dichotomous, defined as any attempt over a lifetime	- Low SES OR 69.27 (CI=10.53–455.84)[a3]
(Park and Lee 2016)	Dichotomous question: question: "Have you attempted suicide during the past 12 months?"	- high OR 5.12 (CI=2.05–12.79)[b1], low OR 9.07 (CI=3.84–21.39)[b1]
(Rim et al. 2020)	Dichotomous question: "Have you attempted suicide during the past 12 months?"	- North Korean-family adolescents: high OR 5.42 (CI=1.07-27.33)[a1], low OR 15.02 (CI=3.22-69.99)[a2]<br>- South Korean-family adolescents: high OR 4.25 (CI=1.15-15.72)[a1], low NS

Abbreviations: NS = not significant. a = multivariate/multiple regression (adjusted). b = confirmed in univariate analysis, no multivariate/multiple analyses (unadjusted). c = only confirmed in univariate analysis, NS in multivariate/multiple analysis; 1 = $p < 0.05$, 2 = $p < 0.01$, 3 = $p < 0.001$

Annex 3. Review findings on completed suicides summarized by individual socioeconomic factor

Authors	Measure of completed suicide	Findings/Strength of association
		**Examined factor: Employment/Occupation**
(Kim et al. 2006)	Gender-specific analysis	- Men: highest OR (>2.0) among Military service, Elementary occupations, Agricultural, forestry and fishery workers, Machine operators and assemblers<br>- Women: Due to the small number of employed women included, no significant differences among women evident
(Kim et al. 2019c)	Gender-and age- specific analysis	- AFF group had highest RR of crude mortality rate for suicide overall followed by the OST, USL, and MNP groups<br>- age specific differences: young women and men (15 -39y) in MNP groups low RR for suicide, markedly higher RR among older individuals
		**Examined factor: Socioeconomic Income/Finances**
(Cho et al. 2007)	Gender- and age- specific analysis	- socioeconomic gradient in suicide not evident when it was analyzed separately in early and late youth<br>- significant socioeconomic gradient when analyzed in total<br>=lower income, higher RR for suicides for boys and girls
(Choi et al. 2019)	Gender-and age- specific analysis	- by sex:<br>• Men: poverty class HR 1.5[a], middle-income class HR 1.31[a]<br>• Women: poverty class HR 1.12[a], middle-income class HR 1.09[a]<br>- by age:<br>• Young-old adults: poverty class HR 1.41[a], middle-income class HR 1.25[a]<br>• Old-old adults: poverty class HR 1.13[a], middle-income class HR 1.13[a]
(Kim et al. 2006)	Gender-and age- specific analysis	- Men: 1,5 – 2 million KWON OR 1.21 (CI=0.85–1.72)[a], 1,2 – 1,5 million KWON OR 1.62 (CI=1.15–2.28)[a], 1 – 1,2 million KWON OR 1.95 (CI=1.39–2.73)[a], ≤ 1 million KWON OR 1.72 (CI=1.23–2.41)[a]<br>- Women: 1,5 – 2 million KWON OR 1.34 (CI=0.40–4.48)[a], 1,2 – 1,5 million KWON OR 1.45 (CI=0.44–4.78)[a], 1 – 1,2 million KWON OR 2.98 (CI= 0.92–9.66)[a], , ≤ 1 million KWON OR 1.51 (CI=0.47–4.88)[a]
(Lee and Hong 2017)	Gender-and age- specific analysis	- suicide rate generally high among individuals in low-income quintiles, people in medical aid group most likely to commit suicide<br>- self-employed at higher risks of committing suicide than wage earners in the same income class<br>- effect of income on suicide is pronounced for males<br>- Effect of income on suicide only marginally influenced by age

Abbreviations: NS = not significant. a = multivariate/multiple regression (adjusted). b = confirmed in univariate analysis, no multivariate/multiple analyses (unadjusted). c = only confirmed in univariate analysis, NS in multivariate/multiple analysis; 1 = $p < 0.05$, 2 = $p < 0.01$, 3 = $p < 0.001$

Authors	Measure of completed suicide	Findings/Strength of association
(Lee et al. 2017)	Gender- and age- specific analysis	- highest HR of suicide found in Medicaid recipients, HR's of suicide increased when income decreased[a1]<br>- Males showed significantly<br>high HRs in almost all income groups, females only significantly high HRs in the Medicaid recipients[a1] group and in the 1st and 4th deciles[a1]<br>- 20-60 years higher HRs of suicide across income groups, adolescent subjects showed a significantly high HR only in the Medicaid recipients group, week effect of income on ≥60 years
(Sohn et al. 2014)	Gender and age-specific analysis	- significantly higher OR's for suicide among adolescents and young adults (<40y)[a]
**Examined factor: Education**		
(Kim et al. 2006)	Gender- specific analysis	- Men: ≤high school OR 1.35 (CI=1.24–1.48)[a], ≤middle school OR 1.36 (CI=1.25–1.48)[a]<br>- Women: ≤high school OR 1.19 (CI=1.03–1.38)[a], ≤middle school OR 1.16 (CI=1.00–1.35)[a]
(Kim et al. 2010)	Gender- specific analysis	- individuals with lower levels of educational attainment exhibited higher rates of suicide<br>- differences in suicide mortality between educational groups grew larger across consecutive years<br>- inequalities were more salient in men than in women
(Lim et al. 2015)	Gender-and age specific analysis	- RR's of college and high school were particularly large, difference between high school and middle school graduate or less was attenuated<br>- increase in inequalities over time (=bigger differences in suicide deaths between educational groups over time)<br>- college education linked to a smaller likelihood respectively risk of committing suicide<br>- mortality rates for suicide in younger women with the lowest education level increased dramatically from 1995 to 2010
**Examined factor: Socioeconomic Place of residency**		
(Chan et al. 2015)	Gender- and age specific analysis	- inverse relationship between the degree of urbanicity and regional suicide rates, more pronounced among women<br>- Increases over time in the suicide rates among youth and working-age adults were greater in large urban centers and in rural regions. For elders, the increase was far greater in rural regions.
(Kim et al. 2006)	Gender-specific analysis	- Men: Metropolitan OR 1.33 (CI=1.22–1.46)[a], Cities OR 1.57 (CI=1.44–1.70)[a], Rural areas OR 1.82 (CI=1.66–1.99)[a]

Abbreviations: NS = not significant. a = multivariate/multiple regression (adjusted). b = confirmed in univariate analysis, no multivariate/multiple analyses (unadjusted). c = only confirmed in univariate analysis, NS in multivariate/multiple analysis; 1 = $p < 0.05$, 2 = $p < 0.01$, 3 = $p < 0.001$

Authors	Measure of completed suicide	Findings/Strength of association
		- Women: Metropolitan OR 1.39 (CI= 1.20–1.62)[a], Cities OR 1.62 (CI= 1.41–1.86)[a], Rural areas OR 2.29 (CI= 1.96–2.66)[a]
(Kim et al. 2010)	Gender- specific analysis	Age-standardized suicide rates (100,000) between 1995 - 2005 highest in rural areas followed by urban and metropolitan areas<br>- consistently positive association between district deprivation and suicides over time
**Examined factor: Socioeconomic Status/Socioeconomic Position/Social class**		
(Kim et al. 2006)	Gender-specific analysis	Men (/class 1): Class 2 OR 1.8 (CI=1.61–2.00)[a], Class 3 OR 2.05 (CI=1.82–2.29)[a], Class 4 OR 2.35 (CI=2.03–2.72)[a]<br>- Women (/class 1): Class 2 OR 1.51 (CI=1.14–1.98)[a], Class 3 OR 2.67 (CI=2.01–3.54)[a], Class 4 OR 2.3 (CI=1.47–3.59)[a]

Abbreviations: NS = not significant. a = multivariate/multiple regression (adjusted). b = confirmed in univariate analysis, no multivariate/multiple analyses (unadjusted). c = only confirmed in univariate analysis, NS in multivariate/multiple analysis; 1 = $p < 0.05$, 2 = $p < 0.01$, 3 = $p < 0.001$

Annex 4. Tabular overview of the aim and main results of the 53 included studies

Author	Aim	Main results
(Ahn et al. 2019)	Evaluation of physical and mental health problems of waged workers with different classes of occupation	- differences in the gender distribution and socioeconomic status of workers in the included types of occupational classes<br>- “Unskilled manual workers” more likely to be older, less educated, have less monthly income, work fewer hours per week and have suicidal ideation
(Bagalkot et al. 2014)	Evaluation of lifetime prevalence of risk factors for suicidal ideation and suicide attempts	- high prevalence of lifetime suicidal behavior in Jeollabuk-do Province is higher than in Korea as a whole<br>- most significant risk factors were found to be social support, family disharmony, anger, depression and low self-esteem
(Chan et al. 2015)	Exploration of geospatial trends of suicide in South Korea from 1992 to 2012	- Suicide rates increased during the study period<br>- Inverse relationship between the degree of urbanicity and regional suicide rates.<br>- Suicide in South Korea as a dynamic process, regional variation linked to migration and urbanization
(Chin et al. 2011)	Investigation of suicidal ideation and its associated factors by sex in adults	- Less SI among those who were married, had higher educational attainment, were non-smokers, were under low stress and had good self-rated health<br>- Effects of stress and depression on SI is higher for men, effects of sleep and subjective body image is higher for women
(Cho et al. 2007)	Examination of possible socioeconomic mortality differences in adolescents	- Clear socioeconomic gradient for all-cause mortality in boys, less evident pattern in girls<br>- transport accidents were the most common cause of death among adolescents<br>- marginally significant socioeconomic difference in suicide mortality for 10–19-year-olds
(Choi et al. 2019)	Identification of the impact of poverty on suicide risk in older adults	- suicide and poverty rates for older adults is highest in South Korea among OECD-Countries<br>- Elderly people living in poverty had a higher risk of suicide than those classed as having a high income<br>- Hazard ratio for suicide risk by income class differed significantly by sex and age group; males and young-old adults aged 60-74 in the poverty group with significantly higher suicide rates compared with females and adults aged 75 or older
(Fukai et al. 2020)	Determination multidimensional (physical, mental, social, spiritual) health status in relation to the presence of depressive symptoms and suicidal ideation in the general population	- Physical, mental, social, and spiritual self-rated health statuses were all found to be associated with an individual’s predisposition to depression and suicidal ideation<br>- Spiritual health status affected depression only in men and mental health status affected depression only in women; social health status in men and mental health status in women were associated with suicidal ideation
(Han and Lee 2013)	Examine whether variation in SI is attributable to administrative level and whether suicidality and	- 2.7% of variation in suicidal ideation was attributable to the administrative area<br>- perceived helpfulness at individual level, organizational participation at administrative-area level associated with suicidal ideation

Abbreviations: NS = not significant. a = multivariate/multiple regression (adjusted). b = confirmed in univariate analysis, no multivariate/multiple analyses (unadjusted). c = only confirmed in univariate analysis, NS in multivariate/multiple analysis; 1 = p < 0.05, 2 = p < 0.01, 3 = p < 0.001

Author	Aim	Main results
	related factors are associated at multiple levels, particularly focusing on social capital	- need of policies who aim preventing suicide at the administrative area and focusing on social capital
(Hong et al. 2011)	Measure of income-related inequalities in depression, suicidal ideation and suicide attempts over a 10-year period (1998-2007)	- persistent pro-rich inequality in depression, suicidal ideation and suicide attempts over the past decade = People with higher incomes were less likely to be affected<br>- inequalities doubled over the time-period of 10 years.<br>- need for expanded social protection policies for the less privileged in the population
(Jeong and Chun 2019)	Identification of individual and regional characteristics that influence suicidal ideation among the elderly population	- age, frequency of communication with friends, religious activity, social activity, leisure activity, trust in neighbors, subjective stress level, depressive symptoms, and subjective health status were significantly associated with suicidal ideation<br>- the lower the regional deprivation level, the higher the suicidal ideation odds ratio<br>- most significant effects found in rural areas.
(Jo et al. 2015)	Investigation of the association between in-school students' part-time work and 1-year suicide attempts	- middle school students' part-time work may increase suicide attempts<br>- no association between part-time work and suicide attempts among high school students
(Joo and Roh 2016)	Evaluation of the risk factors associated with depression and suicide ideation in a rural population	- poor self-rated health, low social support and neurotoxicity positively associated with the risk of depression<br>- low social support, neurotoxicity and farmer's syndrome associated with SI
(Ju et al. 2016)	Investigation of the relationship between socioeconomic status and SI In elderly individuals	- Of 58,900 included participants, 9,265 experienced SI<br>- Participants with food insecurity, low household income, and those who lived alone showed a significant increase in SI and were more likely to experience it
(Kang et al. 2015)	Determination of the prevalence, pattern, and predictors of SI, and suicide attempts of adolescents in the past 12 months	- The prevalence rates for SI and suicide attempts were 19.1% and 4.9%, respectively, among adolescents<br>- Being female, having poor SES and poor perceived academic performance, subjective feelings of depression, cigarette smoking, alcohol use, perceived general medical health, and experience of any sexual intercourse increased the risk for SI and suicide attempts<br>- Suicide attempt rates among Korean female adolescents were highest at age 13 years
(Kang et al. 2014)	Assess the prevalence, incidence, and persistence of SI of elderly adults (≥65y) and to investigate the psychosocial factors associated with these	- Prevalence of SI at baseline, incidence at follow-up, and persistence were 11.5%, 9.6%, and 36.5%<br>- SI at baseline is independently associated with no current employment, lower monthly income, stressful life events, more severe pain, presence of disability, depressive symptoms, and smoking<br>- Incidence of SI is independently predicted by baseline status of unmarried, deficit in social support, severe pain, presence of depressive symptoms, and smoking

Abbreviations: NS = not significant. a = multivariate/multiple regression (adjusted). b = confirmed in univariate analysis, no multivariate/multiple analyses (unadjusted). c = only confirmed in univariate analysis, NS in multivariate/multiple analysis; 1 = $p < 0.05$, 2 = $p < 0.01$, 3 = $p < 0.001$

Author	Aim	Main results
		- Persistent SI is independently predicted by stressful life events and depressive symptoms at baseline
(Kang et al. 2017)	Determination of the relationship between shift work and mental health, particularly insomnia, depression, and SI among electronics production workers	- shift workers had 2.35 greater odds of insomnia, 1.23 of depression and 1.17 of SI<br>- Within the shift worker group, the odds of depression and suicidal ideation increased when respondents had insomnia
(Ki et al. 2017)	Investigation of the pathway from SEP (educational attainment, household income, occupational group) to suicide attempts with attention to potential mediators of individuals	- Most of direct and indirect effects of educational attainment, household income, and occupational group on suicide attempts were significant.<br>- nonemployment status with the largest total and direct effect, educational attainment largest indirect effect<br>- Educational attainment mainly mediated by physical illness and problem drinking, household income and occupational group mainly mediated by anxious or depressed mood and problem drinking
(Kim et al. 2020)	Exploration of changes of job status which influence suicidal risk among workers	- negative change in employment status increased the risk of suicide, but only for males = individuals who are in precarious labor market positions are at greater risk of suicidal thoughts than those in secure labor market positions
(Kim et al. 2016a)	Investigation of the association between SES and suicide attempt	- low education and unemployed young adults had significantly higher rates of attempts<br>- lowest income level associated with significantly higher rates of attempts in only young adult women<br>- Association between SI and suicide attempts was attenuated among those with the lowest and highest income, enhanced among other groups
(Kim and Yoon 2018)	Analysis of the extent to which socioeconomic attainments (education, household income, and employment status) are associated with risk of SI among adults	- Young adults with 2-year colleges and higher, had a lower likelihood of SI<br>- Middle-aged respondents with 4-year colleges and higher had an increased likelihood of SI<br>- Individuals who were not employed had an increased risk of SI<br>- The likelihood of SI, decreased with household income among middle-aged adults
(Kim et al. 2006)	Investigation of the effects of social class on suicide risk	- proportions and OR'S for suicide are higher in young people and for divorced subjects<br>- OR's are higher for residents of rural areas, and people in lower social classes

Abbreviations: NS = not significant. a = multivariate/multiple regression (adjusted). b = confirmed in univariate analysis, no multivariate/multiple analyses (unadjusted). c = only confirmed in univariate analysis, NS in multivariate/multiple analysis; 1 = p < 0.05, 2 = p < 0.01, 3 = p < 0.001

Author	Aim	Main results
(Kim et al. 2016b)	Identify traditional risk factors and investigate risk factors for suicide attempts according to gender	- Trend toward an increase in suicide attempts with decreasing age<br>- Suicide attempts significantly higher among widowed, divorced, or separated men, but lower among women compared with married men<br>- Smoking and depression are risk factors for suicide attempts for both sexes<br>- Monthly household income, myocardial infarction, and osteoporosis associated with suicide attempts for men<br>- Educational level, National Basic Security recipients, family contact, leisure activity, and drinking frequency associated for women
(Kim et al. 2017)	Investigation of the effects of job security on new development of depressive episode, suicide ideation, and decline in self-rated health	- Men with low job security have significantly higher hazard ratios for depression, SI, and worsening self-rated health<br>- Women have significantly higher hazard ratios for depression in the medium and low job security groups
(Kim et al. 2010)	Documentation of socioeconomic inequalities in self-destructive behaviors including suicidal ideation, parasuicide, and completed suicide from 1995-2005	- age-standardized prevalence of suicidal ideation and parasuicide decreased, while completed suicides increased over time in both genders<br>- most prominent increases in completed suicides observed among the elderly<br>- Lower education, rural residence, and area deprivation associated with higher suicide rates.<br>- Absolute and relative inequalities widened over time
(Kim et al. 2014)	Identification of factors associated with suicide ideation in the aged population	- Severity of depression is positively correlated with SI and had the greatest direct impact on SI<br>- Economic status, psychological well-being, and social relationships are negatively correlated with SI<br>- Economic status and social relationships have an indirect influence on SI
(Kim and You 2019)	Examination of the potential of delayed monthly bill payments as a predictor of suicidal behavior in South Korea	- Of 10,988 individuals, 2.7% reported SI and 0.11% reported a suicide attempt in the past year<br>- Adults with two or more late payments had 2.32-fold increased odds of SI and a 10.99-fold increased odds of suicide attempts, compared with individuals who had no late payments

Abbreviations: NS = not significant. a = multivariate/multiple regression (adjusted). b = confirmed in univariate analysis, no multivariate/multiple analyses (unadjusted). c = only confirmed in univariate analysis, NS in multivariate/multiple analysis; 1 = $p < 0.05$, 2 = $p < 0.01$, 3 = $p < 0.001$

Author	Aim	Main results
(Kim et al. 2018)	Investigation the relationship between occupational stress and SI, focusing on gender differences among employees	- All job stresses contributed to increased risk for SI in men and all job stresses except inadequate job control and organizational system were risk factors for SI in women<br>- After adjustment for depression, only inadequate job control and lack of reward remained significant for SI in men, and only interpersonal conflict remained significant in women
(Kim et al. 2019b)	To examine how employment transitions between permanent employment, precarious employment, and unemployment are associated with SI among working employees and whether individuals who transition between these three states are more vulnerable than those who remain	- Compared to the "permanent to permanent" group, individuals in the "permanent to precarious" (OR 1.74), "permanent to unemployment" (OR 1.97), "precarious to precarious" (OR 1.86), and the "precarious to unemployment" (OR 1.43) groups had higher odds of SI<br>- magnitude of such odds was generally higher than that of individuals at annual unemployment or precarious states
(Kim et al. 2019c)	Analysis of suicide and suicide by pesticide rates among workers from 2003-2017 and their associations with occupational groups, and the influence of three major economic indices on these factors	- Age, gender, and time period effects, as well as the effects of macroeconomic indicators, differ by occupational group for suicide and suicide by pesticide → Workers in agriculture, forestry, and fishing showed higher suicide rates than the general population<br>- Suicides from pesticides largely declined in all occupational groups after 2012, when the pesticide ban policy was implemented<br>- real gross domestic product had a positive relationship with suicides overall only in the last five-year period examined in this study; the unemployment rate had a positive relationship across the board
(Ko et al. 2014)	Identification of the relationship of SEP and suicide attempts using both the subjective and objective measures of SEP in the Korean adolescents	- higher risks of suicide attempts were found in the Korean adolescents with the low level of both the subjective and objective SEP in the middle school and with the low subjective SEP and high objective SEP in the high school<br>- subjective SEP is far more important in the high school students than objective SEP
(Kwak and Kim 2017)	Identification of the association between health-related quality of life and mental health by elderly Koreans' occupational status	- Compared to participants employed, those not showed lower health-related quality of life and had more SI<br>- Occupational status significantly affected all of the health-related quality of life dimensions<br>- Manual workers experienced more depression and SI than did non-manual workers
(Lee and Hong 2017)	Investigation of how individual economic status and health conditions (chronic diseases and disabilities) affect the probability of suicidal death	- low economic status significantly increases the probability of suicide<br>- Poor health is strongly related to a high risk of suicide<br>- association between health condition and suicide is more strongly observed among older people than middle-aged individuals
(Lee et al. 2008)	Evaluation of the prevalence and risk factors associated with SI and	- two-week prevalence of SI was 9.8% and the lifetime prevalence of suicide attempts was 3.3%

Abbreviations: NS = not significant. a = multivariate/multiple regression (adjusted). b = confirmed in univariate analysis, no multivariate/multiple analyses (unadjusted). c = only confirmed in univariate analysis, NS in multivariate/multiple analysis; 1 = $p < 0.05$, 2 = $p < 0.01$, 3 = $p < 0.001$

Author	Aim	Main results
	suicide attempts in college students	- most important predictors of SI: depression, probability for bipolar disorder and academic achievement<br>- most important predictor of suicide attempts: socioeconomic status and probability of bipolar disorder
(Lee et al. 2019a)	Develop a risk stratification model for adolescent suicide attempts	- important univariate risk factors for the outcome: dimensional measures of age, sex, breakfast consumption, experience of violence, sleep duration, perceived stress, feeling of sadness, current cigarette smoking, current alcohol drinking, perceived general health, perceived academic record, household economic status and living with biological or adoptive parents
(Lee et al. 2017)	Identification of the relationship between individuals' socioeconomic disposition and suicide risk	- Hazard ratios of suicide showed an increasing trend as socioeconomic position decreased<br>- medicaid recipients had the highest suicide hazard ratio, among them, men had higher hazard ratios than women<br>- Hazard ratios varied across age groups, highest was found 40-59y-group
(Lim et al. 2015)	Examination of the change in the PAF (population attributable fraction, measure to quantify the attributable burden of exposure on a population) of lower educational levels for mortality	- consistent and sharp increase in the attainment of education in the studied time period from 1995-2010 has contributed to the reduction in the PAFs of lower education for mortality<br>- PAFs of suicide in younger females (30–44 years) and of cerebrovascular disease in older males (45–59 years) have increased
(Min et al. 2019)	Comparison of suicidal behaviors between the employed and standardly employed workers	- Persons self-employed, compared with standard workers, were more likely to report SI in both small and middle to large businesses<br>- likelihood for suicide attempt was only significant in persons self-employed in small businesses
(Min et al. 2015)	Comparison of SI and suicide attempts in precarious workers and their non-precarious counterparts	- Employees with precarious work were more likely to exhibit SI and suicide attempts than employees with non-precarious work<br>- Odds were largely attenuated when adjusting for income, education, and depressive feelings → indicates a strong association between suicidal risk and socioeconomic indicators and/or depression
(Moon and Park 2012)	Investigation of the influence of socio-demographic status on SI among middle-aged adults	- SI most prevalent among middle-aged women who engaged in manual labor<br>- Absence of a spouse is significantly associated with suicidal ideation in middle-aged men
(Park and Lee 2015)	Investigation of the age-specific contributions of sociodemographic factors, health status, and health behaviors to SI	- factors associated with SI differ by age<br>- Perceived bad health, stress, and depression had a significant influence on SI in all age groups; depression was strongest predictor among all ages<br>- Disease and marital status did not have a significant effect on SI among those 45–64 years old, education had no effect among those 65 years or older

Abbreviations: NS = not significant. a = multivariate/multiple regression (adjusted). b = confirmed in univariate analysis, no multivariate/multiple analyses (unadjusted). c = only confirmed in univariate analysis, NS in multivariate/multiple analysis; 1 = $p < 0.05$, 2 = $p < 0.01$, 3 = $p < 0.001$

Author	Aim	Main results
(Park et al. 2016a)	Investigation of the risk factors of SI and their PAF (population attributable fraction, measure to quantify the attributable burden of exposure on a population) of the elderly population	- Advanced age and economic status (more than $25,700 per year) were significantly associated with decreased SI risk<br>- Factors that were associated with increased risk were poor social support, sleep problems, chronic illness, poor subjective health, functional impairment, and depression → depression as biggest risk factor for SI
(Park and Lee 2016)	Examination of the factors that affect suicide attempts of adolescents in multicultural families	- risk factors: Female gender, residence in large cities (compared with in rural areas), living with relatives/alone/with friends/in a dormitory or living in a facility (compared with living with family), high and low socio-economic status (compared with a middle-level), high and low academic performance (compared with a middle level), severe perceived stress (compared with non-severe stress), conflicts with a teacher (compared with conflicts with parent), foreign-father/-parent families (compared with foreign-mother family)
(Rim et al. 2020)	Assess the risk of suicide attempts among North Korean adolescents compared to South Korean adolescents, investigate the contributing factors for each group	- significantly higher suicide attempt rate in the North-Korean group<br>- associated with suicide attempts in North Korean adolescents: low or high socioeconomic status, depressive symptoms<br>- associated with suicide attempts in South Korean adolescents: being female, high socioeconomic status, alcohol use, and depressive symptoms
(Ro et al. 2015)	Analysis of factors affecting elderly (≥65y) SI and suicide attempts as well as the paths of these effects	- Depressive symptoms and SI are the only factors that directly affect suicidal attempts<br>- Demographic (income, one person household, job, age, sex), behavioral (alcohol, smoking), and physical activity factors have indirect effects on suicidal attempts → affect suicide attempts through depressive symptoms
(Shin et al. 2015)	Examination the temporal relationship between EHE (Excessive health expenditure) and SI	- 3.4% experienced SI<br>- Recent and greater EHE might increase SI, especially in women
(Shin et al. 2009)	Investigation of predictors of adolescence suicidality in a longitudinal study	- factors of gender, economic status, the overall amount of behavior problems, the tendency to internalizing and externalizing problems, somatic problems, thought problems, delinquent behavior, and aggressive behavior were independent predictors of adolescent SI and self-harm behavior
(Sohn et al. 2014)	Investigation of the difference in medical care expenditure between those who committed suicide from non-illness-related causes and their age- and gender-matched controls	- medical care expenditures increased in only the last 3 months prior to suicide in the adolescent group<br>- significant associations with being a suicide completer and having a rural residence, low socioeconomic status, and high medical care expenditure<br>- After stratification, a significant positive association with medical care expenditures and being a suicide completer was found in the adolescent and young adult groups, no significant results were found in the elderly groups for both men and women

Abbreviations: NS = not significant. a = multivariate/multiple regression (adjusted). b = confirmed in univariate analysis, no multivariate/multiple analyses (unadjusted). c = only confirmed in univariate analysis, NS in multivariate/multiple analysis; 1 = $p < 0.05$, 2 = $p < 0.01$, 3 = $p < 0.001$

Author	Aim	Main results
(Yi and Hong 2020)	Examination of the potential influence of childhood and adult socioeconomic status (SES) on probable depression, SI, and self-esteem among men and women aged 50 years and older	- childhood economic status rarely associated with mental health<br>- Current income consistently was associated with depression, SI and self-esteem in both sexes → mental and psychological health greatly influenced by adult SES<br>- Unemployment is especially significant for SI in men
(Yoon et al. 2015a)	Identification of the effect of long working hours and night/shift work on SI among the employed population	- Employees: SI associated with moderately long WHs (51−60 hours)<br>- Self-employed: SI associated with moderately long and very long WHs (≥60h)<br>- Shift work only significant for female employees
(Yoon et al. 2015b)	Examination of the effect of type D personality and other socio-demographic factors on suicidality in low-income, middle-aged	- Unemployment and absence of spouse as predictors for SI independent of other socioeconomic factors<br>- All type D personality scores predicted higher scores for SI<br>- Association between suicidality and socio-demographic factors (employment or physical health) found only in subjects without type D personality
(Yoon et al. 2016)	Examination of the relationship of high emotional demands and low job control to SI among service and sales workers	- workers who suffered from high emotional demands or low job control more likely to experience SI<br>- high emotional demands in both genders and low job control in men can influence SI
(Yoon et al. 2015c)	Investigation of the relationship between long working hours and suicidal thoughts	- higher OR's for suicidal thoughts for individuals working ≥60h per week<br>- combined effects of long working hours with lower socioeconomic status, or with sleep disturbance, were significantly higher compared to participants who worked less than 52 hours per week with higher socioeconomic status, or with 6–8 hours of nighttime sleep
(Yoon et al. 2017)	Examination of the association between change in employment status and SI among workers	- Compared to those who remained permanent workers, participants who became part-time precarious workers more likely to have SI

Abbreviations: NS = not significant. a = multivariate/multiple regression (adjusted). b = confirmed in univariate analysis, no multivariate/multiple analyses (unadjusted). c = only confirmed in univariate analysis, NS in multivariate/multiple analysis; 1 = $p < 0.05$, 2 = $p < 0.01$, 3 = $p < 0.001$
